# The plasma level and biomarker value of neutrophil gelatinase-associated lipocalin in critically ill patients with acute kidney injury are not affected by continuous venovenous hemofiltration and anticoagulation applied

**DOI:** 10.1186/cc13838

**Published:** 2014-04-22

**Authors:** Louise Schilder, S Azam Nurmohamed, Pieter M ter Wee, Nanne J Paauw, Armand RJ Girbes, Albertus Beishuizen, Robert HJ Beelen, AB Johan Groeneveld

**Affiliations:** 1 Department of Nephrology, VU University Medical Center, De Boelelaan 1117, 1081 HV, Amsterdam, The Netherlands; 2 Department of Molecular Cell Biology and Immunology, VU University Medical Center, Amsterdam, The Netherlands; 3 Department of Intensive Care, VU University Medical Center, Amsterdam, The Netherlands; 4 Department of Intensive Care, Erasmus Medical Center, Rotterdam, The Netherlands

## Abstract

**Introduction:**

Neutrophil gelatinase-associated lipocalin (NGAL) is a biomarker of acute kidney injury (AKI), and levels reflect severity of disease in critically ill patients. However, continuous venovenous hemofiltration (CVVH) may affect plasma levels by clearance or release of NGAL by activated neutrophils in the filter, dependent on the anticoagulation regimen applied. We therefore studied handling of NGAL by CVVH in patients with AKI.

**Methods:**

Immediately before initiation of CVVH, prefilter blood was drawn. After 10, 60, 180, and 720 minutes of CVVH, samples were collected from pre- and postfilter (in- and outlet) blood and ultrafiltrate. CVVH with the following anticoagulation regimens was studied: no anticoagulation in case of a high bleeding tendency (*n* = 13), unfractionated heparin (*n* = 8), or trisodium citrate (*n* = 21). NGAL levels were determined with enzyme-linked immunosorbent assay (ELISA).

**Results:**

Concentrations of NGAL at inlet and outlet were similar, and concentrations did not change over time in any of the anticoagulation groups; thus no net removal or production of NGAL occurred. Concentrations of NGAL at inlet correlated with disease severity at initiation of CVVH and at the end of a CVVH run. Concentrations of NGAL in the ultrafiltrate were lower with citrate-based CVVH (*P* = 0.03) and decreased over time, irrespective of anticoagulation administered (*P* < 0.001). The sieving coefficient and clearance of NGAL were low and decreased over time (*P* < 0.001).

**Conclusions:**

The plasma level and biomarker value of NGAL in critically ill patients with AKI are not affected by CVVH, because clearance by the filter was low. Furthermore, no evidence exists for intrafilter release of NGAL by neutrophils, irrespective of the anticoagulation method applied.

## Introduction

Acute kidney injury (AKI) is common in critically ill patients, particularly in case of sepsis, and associated with increased morbidity and mortality, even in the long term [1]. The diagnosis of AKI heavily relies on serum creatinine (sCr) and urinary output, but these markers are imperfect in non-steady-state conditions such as AKI. In patients with subclinical AKI, further damage may be prevented by appropriate measures. Therefore, biomarkers to predict AKI before the increase of sCr have been studied intensively. Neutrophilic gelatinase-associated lipocalin (NGAL) is a small, 25-kDa protein and one of the neutrophil secondary granule proteins [2]. It is also rapidly induced in distal tubular segments of injured nephrons with stress [3]. Therefore, urinary and plasma levels are helpful in early prediction of AKI [4-7], in prediction of severity of AKI [6-9] and AKI-related outcomes, such as the need for renal-replacement therapy (RRT), as well as mortality [5-7,9-12]. Also, levels of NGAL are associated with disease severity, and its levels are more profoundly elevated during sepsis [5,7,11,13-15]. However, it remains to be elucidated whether continuous venovenous hemofiltration (CVVH) in the treatment of AKI affects plasma NGAL levels and thus the biomarker value, either by clearance, adsorption, or production of NGAL in the filter. One study described removal of NGAL by filtration and possibly adsorption *in vitro*[16]. A small *in vivo* series (*n* = 3) reported on NGAL levels in the ultrafiltrate of patients with AKI taking citrate-based CVVH; the clearance of NGAL was low, and the inlet concentration of NGAL over 24 hours of CVVH did not decline [17].

However, plasma NGAL may decline after just one session of hemodialysis [18]. Conversely, release of NGAL by neutrophils has been reported in extracorporeal circulation during cardiac surgery [19]. Additionally, the anticoagulation regimen applied during CVVH could influence release by neutrophils and handling of NGAL by the filter and extracorporeal circuit. Moreover, it has been postulated that citrate prevents degranulation of neutrophils induced by heparin, complement activation, and blood-filter contact during CVVH, partly by chelating calcium, necessary for neutrophil degranulation [20,21].

The aim of the current study was to assess whether NGAL is cleared by CVVH sufficiently to affect its plasma levels and biomarker value in critically ill patients with AKI. We further hypothesize that citrate-based CVVH diminishes release of NGAL by neutrophils in the extracorporeal circuit and filter.

## Materials and methods

The patient population and methods for this study are reported elsewhere [22,23]. Patients for this companion study were recruited during office hours for sampling when participating in one of the two following prospective studies. The first study started 1 year before the availability of a custom-made citrate-based replacement fluid. From March 2004 to September 2005, patients admitted to the intensive care unit (ICU) who developed AKI necessitating CVVH, but in whom heparin was contraindicated because of a high bleeding tendency, were either treated by anticoagulant-free CVVH (*n* = 13) or, after becoming available in 2005, by regional citrate anticoagulation (*n* = 10) and were prospectively monitored. High risk for bleeding was arbitrarily defined as a platelet count of less than 40 × 10^9^/L, an activated partial thromboplastin time (aPTT) of more than 60 seconds, a prothrombin time of more than 2.0 international normalized ratio, recent major bleeding, or active bleeding. Because all patients were treated according to local standards, the need for informed consent was waived for this study.

Patients were also recruited from a second study, a multicentered randomized controlled trial initiated in 2006 (the so-called Citrate Anticoagulation versus Systemic Heparinization, CASH trial, clinicaltrials.gov number NCT00209378). Patients admitted to the ICU who developed AKI necessitating CVVH without a high bleeding risk, were randomized between unfractionated heparin as anticoagulant (*n* = 8) or citrate (*n* = 11). Only patients from our hospital were studied for this companion study. Informed consent was obtained from all study participants or their next-of-kin. Study protocols were approved by the local medical ethical committee (VU University Medical Ethical Committee) and performed in accordance with the Declaration of Helsinki. We pooled the studies because of the mechanistic nature of the current article, without focus on patient-centered outcomes.

### Treatment protocol

The indication for CVVH was based on standard clinical criteria that include AKI accompanied by hemodynamic instability, ongoing hypercatabolism, diuretic-resistant fluid overload, respiratory distress, multiorgan failure, or some combinations of these factors. CVVH was performed by using a hemofiltration machine (DIAPACT; B. Braun Medical, Melsungen, Germany). Vascular access was obtained by the insertion of an 11 F double-lumen catheter (GAMCATH; Gambro, Hechingen, Germany) into the femoral, subclavian, or jugular vein. A 1.9 m^2^ highly permeable cellulose triacetate filter (NIPRO UF-205; Nissho Corp., Osaka, Japan, cut-off approximately 40 kDa) was used in all treatments. For lactate- or bicarbonate-based CVVH, commercially prepared buffer solutions were used (BH504 or HF32bic, respectively; Dirinco, Rosmalen, The Netherlands). Patients with high serum lactate levels (>5 m*M*) were routinely treated with bicarbonate-buffered rather than lactate-buffered CVVH. For the use of citrate, a replacement solution was custom-made by Dirinco (Rosmalen, The Netherlands). Blood-flow rate was set at 180 ml/min in all groups. Replacement fluid was administered at a standard rate of 2,000 ml/h in the patients receiving no anticoagulation or heparin with bicarbonate or lactate-containing replacement fluids. Replacement-fluid rate in the citrate group was set at 2,400 ml/h, and, in all cases, replacement fluids were infused in predilutional mode. Patients taking heparin were administered a heparin bolus of 5,000 IU followed by a body weight-based continuous infusion targeting an aPTT between 45 and 55 seconds. Patients receiving citrate-based therapy had a separate intravenous infusion with calcium glubionate (Calcium Sandoz, containing 0.225 m*M* calcium; Novartis Consumer Health, Breda, The Netherlands). Calcium administration was adapted to concentrations of ionized calcium in the patient by an especially designed algorithm, as described before [22]. The target ionized calcium concentration in the circuit was 0.3 m*M*, but not routinely monitored, because this is almost uniformly achieved with the aforementioned settings [22].

### Study protocol

At inclusion, demographic variables were recorded, such as age, gender, and reason for ICU admission. Assessment of disease severity on ICU admission was done according to the Acute Physiology And Chronic Health Evaluation II (APACHE II), the Simplified Acute Physiology Score II (SAPS II), and the Sequential Organ Failure Assessment score (SOFA). Patients had not yet received renal-replacement therapy previously, and in 40 (96%) of 42 patients, the first filter run was studied; in two patients, a later filter run was studied because of technical reasons. We collected systemic inflammatory response syndrome (SIRS) criteria: body temperature >38°C; a heart rate of >90 beats/min; a respiratory rate of >20 breaths/min or mechanical ventilation; and white blood cell counts of <4.0 × 10^9^/L or >12.0 × 10^9^/L. When SIRS (two or more criteria) and an infection were present (either clinically suspected or microbiologically confirmed), patients were classified as having sepsis. Venous blood samples were collected from the hemofiltration catheter from each patient before the initiation of CVVH and administration of heparin. Heparin was given immediately after filter connection. Thereafter, blood samples were collected at 10, 60, 180, and 720 minutes from the pre- and postfilter pole, or until the filter terminated, if occurring within 720 minutes. A time period up to 720 minutes was chosen because we expected clearance of NGAL to occur early after initiation of CVVH before filter saturation occurred. Prefilter blood was invariably drawn before addition of replacement fluids, and thus, reported concentrations of NGAL at inlet are not diluted. Leukocytes, platelets, and serum creatinine concentrations were measured before initiation of CVVH and routinely thereafter. In all patients, a zero fluid balance was achieved during the time points at which blood samples were drawn. Ultrafiltrate samples were collected from the appropriate ports. Samples were collected in standard Vacutainer tubes (Becton Dickinson, Erembodegem, Belgium) with ethylenediaminetetraacetic acid (EDTA), benzamidine, and soybean trypsin inhibitor added. All samples were centrifuged at 1,300 *g* for 10 minutes at 4°C and stored at −80°C until assayed in 2009.

### Measurements and calculations

NGAL concentrations were measured with enzyme-linked immunosorbent assays (ELISAs). Commercially available antibody duosets were used (R&D Systems, UK, Lipocalin-2/NGAL Duoset, DY1757). Plasma samples and ultrafiltrate were measured in separate assays with NGAL standards prepared in appropriate matrix solutions, 0.5% bovine albumin serum in phosphate-buffered saline or fresh ultrafiltrate. All measurements were done according to the protocols provided by the manufacturer. The lower detection limit of NGAL was approximately 30 ng/ml. Each sample was run in duplicate, and the mean concentration was calculated. Formulas used to evaluate fluxes are described in the following list:

### Formulas used to evaluate fluxes

Q_i_ = Q_b_ × (1-Ht), Q_o_ = Q_i_

M_i_ = Q_i_ × Ci

M_o_ = Q_o_ × C_o_

M_uf_ = Q_uf_ × C_uf_

M_tr_ = M_i_ - M_o_

M_ad_ = M_tr_ - M_uf_

C = Ci × Q_i_/(Q_i_ + RF)

SC = 2 × C_uf_/(C + C_o_)

Abbreviations

Ci, Concentration in inlet plasma before addition of replacement fluid (ng/ml)

C_o_, Concentration in outlet plasma (ng/ml)

C_uf_, Concentration in ultrafiltrate (ng/ml)

Q_b_, Inlet blood-flow rate (ml/min)

Q_i_, Inlet plasma-flow rate (ml/min)

Q_o_, Outlet plasma-flow rate (ml/min)

Q_uf_, Ultrafiltration flow rate (ml/min)

M_i_, Mass inlet rate (ng/min)

M_o_, Mass outlet rate (ng/min)

M_uf,_ Mass ultrafiltration rate (ng/min)

M_tr_, Mass removal rate (ng/min)

M_ad_, Mass adsorption rate (ng/min)

C, Concentration in inlet plasma after addition of replacement fluid (ng/ml)

RF, Replacement fluid-flow rate (ml/min)

SC, Sieving coefficient

### Statistical analysis

Because of mostly non-gaussian distributions, data are presented as median and range. No baseline differences were found between the two separate citrate groups (citrate versus no-anticoagulation and citrate versus heparin), so the citrate data were pooled. When appropriate, data were log-transformed to achieve normal distributions. The values for the total mass-production rate and mass-adsorption rate were ranked, because some values were negative and could not be log-transformed. Group differences were evaluated by using χ^2^ or Kruskal-Wallis tests, where appropriate. To evaluate differences according to anticoagulation regimens in time, we used generalized estimating equations (GEEs), taking repeated measurements in the same patient into account. The focus of GEEs is on estimating differences between anticoagulation groups and time points, and their first-order interaction (that is, differences between anticoagulation groups over time), and associated *P* values are reported. Spearman correlation coefficients were used to express relations. A *P* < 0.05 was considered statistically significant. Exact *P* values are given unless <0.001.

## Results

### Patient characteristics

At baseline, all patients included in this study (*n* = 42) met the criteria for SIRS, and 18 (43%) of 42 patients met the criteria for sepsis. The no-anticoagulation group (*n* = 13) had higher SAPS II and SOFA scores than did the other groups. The creatinine concentration at baseline was lower in the no-anticoagulation group than in the other groups, as were platelet levels. The prescribed dose was 22 (16 to 32) ml/kg/h in the no-anticoagulation group, 22 (11 to 32) ml/kg/h in the heparin group, and 23 (16 to 31) ml/kg/h in the citrate group (*P* = 0.68). No off time and no net fluid removal occurred during the study period, so the delivered dose equaled the prescribed dose. The median survival time of the filter was 10 (3 to 66) hours in the no-anticoagulation group, 17 (1 to 70) hours in the heparin group, and 45 (five to 138) in the citrate group (*P* = 0.03). In 13 (31%) of 42 patients, the filter terminated within 720 minutes; seven (54%) of 13 in the no-anticoagulation group, three (38%) of eight in the heparin group, and three (14%) of 21 in the citrate group (*P* < 0.001) (Table 1).

**Table 1:** Patient characteristics

	**No anticoagulation**	**Heparin**	**Citrate**	
	***n*** **= 13**	***n*** **= 8**	***n*** **= 21**	***P***
Sex, male	7 (54)	6 (75)	14 (75)	0.59
Age, years	70 (34–84)	57 (23–81)	64 (32–84)	0.49
Weight, kg	70 (50–100)	74 (55–135)	78 (60–110)	0.41
Reason for admission				0.29
Respiratory	2 (15)	5 (63)	7 (33)	
Circulatory	3 (23)	1 (13)	4 (19)	
Trauma	2 (15)	0	1 (5)	
Post-CPR	2 (15)	0	2 (10)	
Postoperative	4 (31)	1 (13)	7 (33)	
AKI	0	1 (13)	0	
Vasopressor dependent	13 (100)	6 (75)	16 (76)	0.15
Mechanical ventilation	11(85)	8 (100)	19 (91)	0.51
Sepsis	5 (39)	5 (63)	8 (38)	0.46
APACHE II at ICU admission	28 (11–42)	22 (15–37)	25 (14–41)	0.47
SAPS II at ICU admission	75 (43–112)	47 (37–77)	52 (32–86)	0.002
SOFA at ICU admission	14 (7–21)	11 (8–15)	13 (8–18)	0.02
ICU stay, days	5 (2–82)	20 (4–48)	20 (3–81)	0.08
LOS in ICU at start CVVH, days	1 (1–5)	2 (1–4)	2 (0–14)	0.74
**Biochemical data**				
Creatinine at start, μ*Μ*	249 (100–410)	420 (156–626)	326 (47–622)	0.01
Creatinine 24 hours, μ*M*	206 (140–250)	284 (125–463)	232 (46–441)	0.05
Urea at start, m*M*	21 (6–48)	32 (12–97)	21 (6–41)	0.25
Urea 24 hours, m*M*	17 (6–34)	17 (11–102)	14 (6–28)	0.24
Leukocytes at start, ×10^9^	8 (1–17)	12 (7–20)	12 (1–26)	0.29
Leukocytes 24 hours, ×10^9^	10 (1–26)	13 (6–26)	11 (2–28)	0.85
Platelets at start, ×10^9^	65 (22–173)	167 (44–352)	118 (35–332)	0.03
Platelets 24 hours, ×10^9^	59 (13–148)	117 (27–239)	105 (5–349)	0.02

Median (and range) or number (percentage). AKI, acute kidney injury; APACHE II, Acute Physiology and Chronic Health Evaluation II; CRP, cardiopulmonary resuscitation; ICU, intensive care unit; LOS, length of stay; SAPS II, Simplified Acute Physiology Score II; SOFA, Sequential Organ Failure Assessment score.

**Figure 1:**
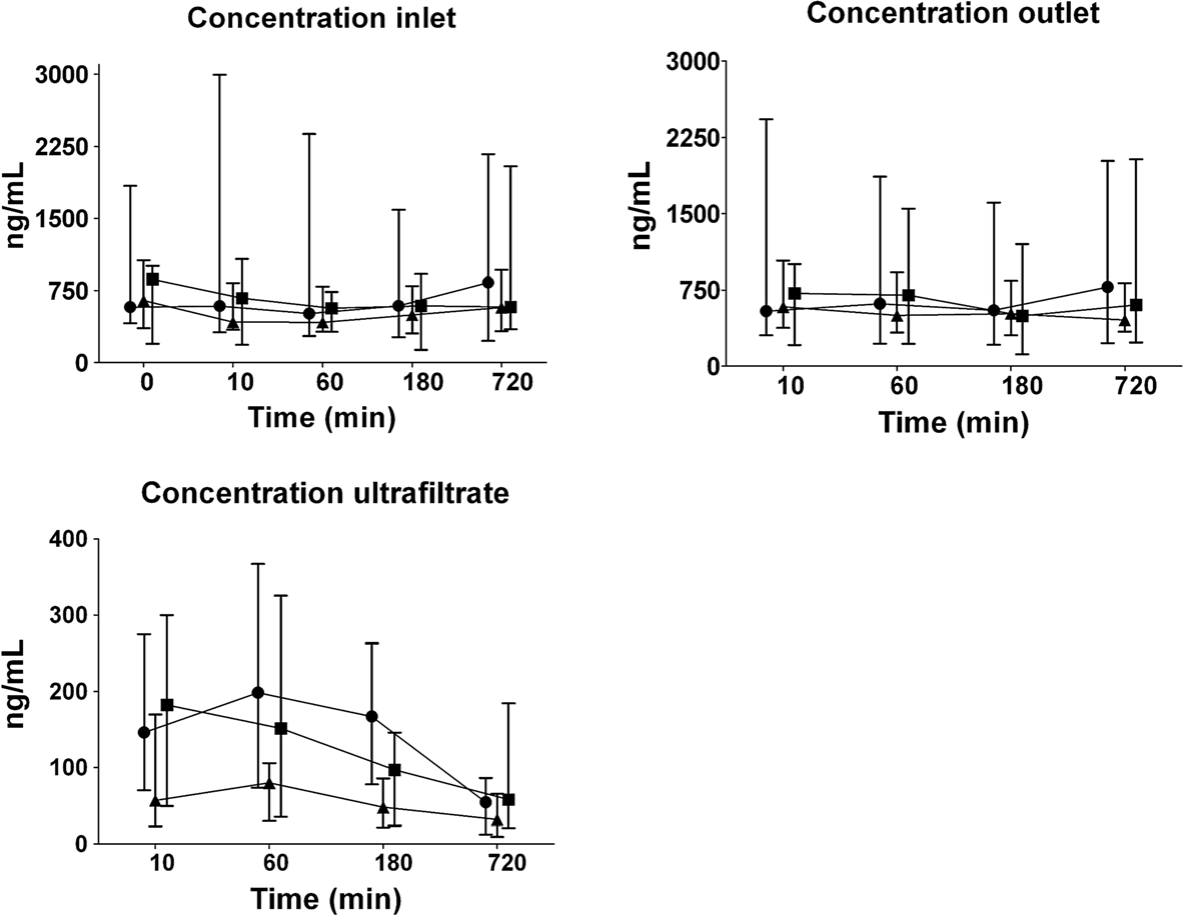
**Concentration of NGAL during CVVH over time (median and interquartile range) at inlet (before addition of replacement fluid), outlet, and ultrafiltrate.** Symbols: ● no anticoagulation, ■ heparin, ▲citrate. Levels of NGAL at inlet and outlet were similar across groups and did not change over time. Levels of NGAL in the ultrafiltrate were lower in the citrate group (*P* = 0.03) and decreased over time in all groups (*P* < 0.001).

**Figure 2:**
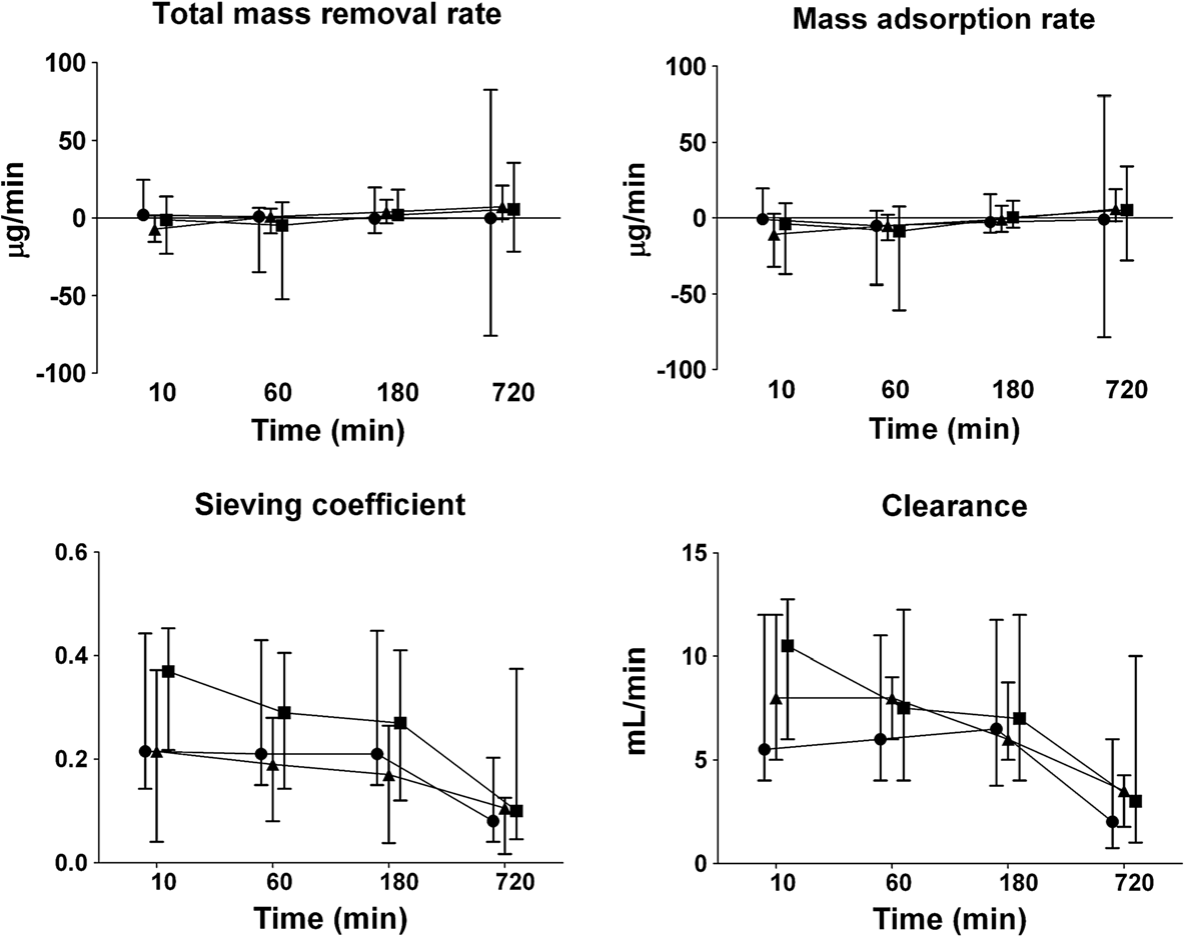
**Mass removal rate, mass adsorption rate, sieving coefficient, and clearance of NGAL during CVVH over time (median and interquartile range).** Symbols: ● no anticoagulation, ■ heparin, ▲citrate. Total mass-removal rate and mass-adsorption rate were similar across groups and did not change over time. Sieving coefficient and clearance decreased over time in all groups (*P* < 0.001).

NGAL was detectable in all plasma and ultrafiltration samples. The concentrations of NGAL at inlet, outlet, and in the ultrafiltrate over time during CVVH are presented in Figure 1. Concentrations at inlet and outlet were similar and did not change over time in any of the anticoagulation groups. Concentrations of NGAL in the ultrafiltrate were lower in the citrate group (*P* = 0.03) and decreased over time in all groups (*P* < 0.001). The total mass removal rate and total mass adsorption rate did not differ between groups and remained unchanged over time (Figure 2). The sieving coefficient was low and did not differ between groups, with medians ranging from 0.2 to 0.4. The sieving coefficient decreased over time in all groups (*P* < 0.001). Also, the clearance of NGAL, calculated from sieving coefficient times ultrafiltration rate (corrected for predilution), was similar in groups and decreased over time in all groups (*P* < 0.001); from 6 (2 to 44) to 2 (0 to 6) ml/min in the no-anticoagulation group, from 10 (four to 18) to 3 (0 to 16) ml/min for heparin and from 8 (0 to 40) to 4 (1 to 9) ml/min in citrate-based CVVH. Results of an analysis excluding all patients in whom sample collection could not be completed because of premature filter termination did not differ from those of an analysis of all patients (data not shown).

### Correlations

No correlation was noted between NGAL and creatinine levels at initiation of CVVH (*P* = 0.53). Plasma levels of NGAL at inlet at the start of CVVH correlated with those at the end of the CVVH run (*r*_s_ = 0.88, *P* < 0.001). Also, plasma levels of NGAL at inlet at initiation of CVVH correlated with APACHE II scores (*r*_s_ = 0.30, *P* = 0.05), SAPS II scores (*r*_s_ = 0.36, *P* = 0.03) and SOFA scores (*r*_s_ = 0.32, *P* = 0.04) at admission, and plasma levels of NGAL at the end of the CVVH run persistently correlated with these disease-severity scores (for APACHE II, *r*_s_ = 0.35, *P* = 0.03, for SAPS II, *r*_s_ = 0.28, *P* = 0.08, and for SOFA, *r*_s_ = 0.33, *P* = 0.04).

### Sepsis and mortality

When analyzing all measured concentrations at inlet, concentrations of NGAL did not differ between patients with sepsis (879 (77 to 9,070) ng/ml) and patients without sepsis (544 (56 to 2,460), *P* = 0.14). Sixteen (38%) of the 42 patients died in the ICU. There was a trend for higher plasma levels of NGAL at inlet in nonsurvivors at initiation of CVVH; 1,035 (121 to 9,070) ng/ml versus 607 (56 to 3,950) ng/ml in survivors (*P* = 0.11). This trend persisted at the end of the CVVH run; 896 (44 to 4,370) ng/ml versus 451 (91 to 4,640) ng/ml in survivors (*P* = 0.07), because no decrease in NGAL was noted over time (*P* = 0.32).

## Discussion

The present study shows that plasma NGAL levels in critically ill patients with AKI are not affected by CVVH, irrespective of the anticoagulation applied.

Among biomarkers for early detection of AKI and its severity, NGAL is probably most studied, whereas predictive values vary among studies [9,10]. Plasma levels are usually more elevated with increasing disease severity, in patients with sepsis (versus nonsepsis), and in those destined to die, even during RRT [5-7,9-12]. Conversely, low values may help predict recovery from AKI after discontinuation of RRT [12]. Because CVVH could influence plasma levels of NGAL and thus its predictive value, by clearance or by production of NGAL in the filter, we studied handling of NGAL in patients receiving CVVH.

Concentrations of NGAL at inlet before addition of replacement fluid, representing patient’s plasma levels, did not change over time and correlated with disease severity irrespective of a CVVH run. Our results are in concordance with a small series of patients (*n* = 3) with AKI taking citrate-based CVVH, where inlet concentrations of NGAL over 24 hours of RRT did not decline [17]. Four patients had plasma NGAL levels at initiation of CVVH of <150 ng/ml, which is low, given the reported cut-off values of approximately 150 ng/ml for predictive models of AKI [6,8,13]. This illustrates that clinical assessment can override the predictive value of NGAL in deciding on initiation of CVVH.

Concentrations of NGAL measured at the outlet were similar to those at the inlet in all groups, suggesting that no net removal or production of NGAL occurred during CVVH. Overall, the sieving coefficient and clearance of NGAL were lower than expected, based on its molecular size, yet in concordance with results of others [16,17]. By binding to other molecules, NGAL may exceed size limits for passage through the filter. It also seems possible that adsorption contributed to a low sieving coefficient, even though calculated adsorption rates were very low. However, neither low clearance nor absorption resulted in net removal of NGAL during CVVH, so that we cannot formally exclude concomitant production, for instance via release by activated neutrophils. Moreover, any production in the filter could be offset by adsorption.

We cannot judge the inhibitory effect of citrate on neutrophil activation in this respect, because we did not observe net production of NGAL in the filter in any of the anticoagulation groups. Concentrations of NGAL in the ultrafiltrate were lowest in the citrate group, but this could partly be explained by dilution due to higher ultrafiltration rates. Dissimilar degranulation patterns of primary and secondary granules of neutrophils might account for the absence of NGAL release in the filter, because primary granular markers elastase and myeloperoxidase (MPO) are released during heparin-CVVH, whereas secondary granular NGAL apparently is not [20]. Others observed that there was lower release of lactoferrin, a secondary granular product similar to NGAL, than of MPO during citrate-based dialysis [24].

The limitations of the present study include the relatively small size of groups and the absence in part of randomization, explaining some baseline differences among the groups. Patients in the no-anticoagulation group did not receive anticoagulation because of a bleeding tendency and were more severely ill, demonstrated by higher SAPS II and SOFA scores at baseline and lower platelet counts. We cannot exclude earlier start of CVVH in the no-anticoagulation group than in the other groups, because initial creatinine was lower, but this does not invalidate our conclusions of this pathophysiologic study. We have evaluated the course of study variables for up to 12 hours during a single filter run only and do not exclude changes beyond that time interval or subsequent CVVH runs.

## Conclusions

The biomarker value of NGAL in critically ill patients with AKI are not affected by CVVH, because clearance by the filter was low. Therefore, plasma levels of NGAL may be used for prognostication in patients receiving CVVH. Furthermore, probably no intrafilter release of NGAL by neutrophils occurs, irrespective of the anticoagulation method applied.

## Key messages

● Plasma levels of NGAL in critically ill patients with acute kidney injury correlate with disease severity at initiation of CVVH and at the end of the a CVVH run

● No net removal of NGAL is found during CVVH, and therefore plasma levels of NGAL may be used for prognostication

● No evidence exists for production of NGAL in the filter by neutrophils, irrespective of the type of anticoagulation applied

## Abbreviations

AKI: Acute kidney injury; APACHE II: Acute Physiology And Chronic Health Evaluation II; aPTT: activated partial thromboplastin time; CVVH: continuous venovenous hemofiltration; EDTA: ethylenediaminetetraacetic acid; ELISA: enzyme-linked immunosorbent assay; GEE: generalized estimating equation; ICU: intensive care unit; MPO: myeloperoxidase; NGAL: neutrophil gelatinase-associated lipocalin; RRT: replacement therapy; SAPSII: Simplified Acute Physiology Score; sCr: serum creatinine; SIRS: systemic inflammatory response syndrome; SOFA: Sequential Organ Failure Assessment score.

## Competing interests

The authors declare that they have no competing interests.

## Authors’ contributions

LS: conception and design, data collection and analysis, manuscript writing, and final approval of the manuscript. SN: data collection and analysis, critical revision, and final approval of the manuscript. NP: data analysis, critical revision, and final approval of the manuscript. RB: data analysis, critical revision, and final approval of the manuscript. AB: data collection, critical revision, and final approval of the manuscript. ARG: data collection, critical revision, and final approval of manuscript. PW: conception and design, critical revision, and final approval of the manuscript. ABG: conception and design, data collection and analysis, manuscript writing, and final approval of the manuscript. All authors read and approved the final manuscript.
